# The physiological responses to volume-matched high-intensity functional training protocols with varied time domains

**DOI:** 10.3389/fphys.2024.1511961

**Published:** 2025-02-11

**Authors:** Jessica S. Smith, Gabriella F. Bellissimo, Fabiano T. Amorim

**Affiliations:** ^1^ Department of Sport and Exercise Sciences, State University of New York at Oneonta, Oneonta, NY, United States; ^2^ Exercise Physiology Laboratory, Department of Health, Exercise, and Sports Sciences, University of New Mexico, Albuquerque, NM, United States; ^3^ Department of Health and Human Performance, College of Idaho, Caldwell, ID, United States

**Keywords:** cardiorespiratory fitness, muscular fitness, resistance training, high-intensity interval training, cross-training, CrossFit

## Abstract

**Background:**

High-intensity functional training (HIFT) is typically performed with minimal or no rest periods, including “rounds for time” (RFT) or “as many rounds or repetitions as possible” (AMRAP) design. Alternatively, some HIFT workouts can be performed with prescribed rest intervals (e.g., “every minute on the minute” [EMOM]) that may have significant effects on physiological responses.

**Purpose:**

To compare the physiological responses between two different HIFT workouts (EMOM and RFT) that were matched for total work volume (TWV).

**Methods:**

Twelve trained individuals (six males and six females) performed two HIFT protocols, EMOM and RFT. Both the EMOM and RFT included five rounds of five power cleans, eight kipping pull-ups, six dumbbell thrusters, and ten burpees performed in this order. Measurements of heart rate (HR), oxygen consumption (VO_2_), rating of perceived exertion (RPE) (1–10 scale), blood lactate (BLA), creatine kinase (CK), excess post-exercise oxygen consumption (EPOC), and muscle oxygen saturation (SmO_2_) were performed.

**Results:**

Time domains were significantly different for the EMOM and RFT workouts (20 vs. 12 min ± 3 min, *p* < 0.00). There were significant differences between the EMOM and RFT for HR (153 ± 19 bpm vs. 171 ± 12 bpm, *p* < 0.01), VO_2_ (30.8 ± 3 mL/kg/min vs. 38.1 ± 5 mL/kg/min, *p* < 0.00), RPE (4 ± 1 vs. 7 ± 1, *p* < 0.00), and EPOC-AUC (3.5 ± 1.2 mL/kg/min vs. 5.0 ± 1.3 mL/kg/min, *p* < 0.00); however, there were no significant differences in mean SmO_2_ (*p* = 0.44). An interaction effect revealed that BLA was lower for the EMOM (6.5 ± 2.7 mmol/L) than the RFT (11.2 ± 2.1 mmol/L) post-exercise (*p* < 0.00). Conversely, there was no interaction effect for CK (*p* < 0.16), yet a significant increase was observed from pre- to post-exercise for both the EMOM and the RFT (*p* < 0.01).

**Conclusion:**

The RFT induced greater physiological stress than the EMOM, indicating that prescribed rest intervals significantly affect the metabolic, cardiovascular, and perceptual responses during high-intensity functional exercise. Furthermore, the RFT may provide a greater cardiorespiratory stimulus, while the EMOM may be more suitable for technique development and recovery in trained individuals.

## Introduction

The fitness industry has evolved from traditional resistance and endurance exercise programs to combined, time-efficient programs that address cardiorespiratory and muscular fitness components in a single exercise session (Cosgrove et al., 2019; Kliszczewicz et al., 2021). One example is high-intensity functional training (HIFT), which involves functional, multi-joint exercises performed at high intensity that are designed to improve health- and skill-related components of fitness (Feito et al., 2018). This type of functional training incorporates endurance and resistance exercises that engage whole-body motor recruitment patterns across multiple planes of movement, such as running, biking, rowing, squats, deadlifts, pull-ups, cleans, snatches, and jumps (Feito et al., 2018). The level of exercise intensity or metabolic stress is determined by the combination of work volume, load, set duration, rest interval duration, and exercise selection, which will influence the magnitude of physiological responses (Haltom et al., 1999). More specifically, HIFT sessions performed with short rest periods are potent stimuli for metabolic and cardiovascular responses (Feito et al., 2018; Jacob et al., 2020).

High-intensity functional training incorporates multi-joint movements and improves ten fitness domains, including cardiorespiratory endurance, stamina, strength, flexibility, power, speed, coordination, agility, balance, and accuracy (Feito et al., 2019). A HIFT workout may include elements of gymnastics (e.g., handstand and ring exercises), weightlifting derivatives (e.g., barbell squats and presses), and cardiovascular endurance exercises (e.g., running or rowing) (Fisker et al., 2017; Feito et al., 2018), generally performed in quick, successive repetition, with little to no recovery (Glassman, 2007). Typically, workouts are designed to perform the exercises continuously with the goal to complete a set volume in the shortest duration possible, “rounds for time” (RFT), or to perform “as many rounds or repetitions as possible” (AMRAP) in a set time domain (Jacob et al., 2020). Other workouts may be designed in an interval format (prescribed rest periods) for a set time with varied volume or a set volume with varied time to completion.

Research has demonstrated significant differences among various HIFT workouts, all of which are generally characterized as high intensity (McDougle et al., 2023). However, few studies have directly compared HIFT protocols that are matched for either volume or time domain. A study by Toledo et al. (2021) compared blood lactate, heart rate (HR), and training load (session rating of perceived exertion [RPE] + total training time) in two different HIFT workouts, RFT and AMRAP, with similar total volumes in trained men and women. Heart rate response was not different between workouts. The greatest responses were found in the RFT compared to the AMRAP for RPE, training load, and maximum repetitions completed for prescribed exercises in women and lactate in men. In another study, Hernández-Lougedo et al. (2021) compared two protocols that both included an RFT component. One of the protocols was adapted to include a 1-min rest interval after each round of exercise. The final HR and average HR were not different between the workouts; however, lower RPE and lactate were observed in the adapted protocol. Regarding time domains, Butcher et al. (2015) compared HR and RPE responses to two different CrossFit^®^ sessions (interval-based and AMRAP) with matched time domains. They found that RPE was similar in both exercise sessions; however, HR responses to the interval-based session were approximately 10% lower than during the AMRAP session, despite the work during the interval-based session being conducted at an “all-out” effort.

HIFT is implemented by novice trainees seeking health and fitness improvements, as well as trained athletes who utilize HIFT as a cross-training program or for competition in functional fitness events. With the worldwide rise in popularity over the past decade, HIFT has established itself as a significant niche within the fitness industry (Feito et al., 2019). Thus, further research characterizing different workout designs within HIFT is necessary to understand the specific physiological responses and associated adaptations, which will provide valuable insights for future exercise prescription and program design. Currently, no studies have compared volume-matched, “every minute on the minute” (EMOM) and RFT designs. Therefore, the first aim of the present study was to compare the differences in the physiological responses between two HIFT workouts that are identical for exercise selection, repetition scheme, and volume yet differ in design: continuous-based (RFT) with varied time domain and interval-based with set time domain (EMOM). We hypothesized that the RFT would induce greater metabolic, cardiovascular, and perceptual responses than the EMOM.

## Materials and methods

### Study design

**FIGURE 1:**
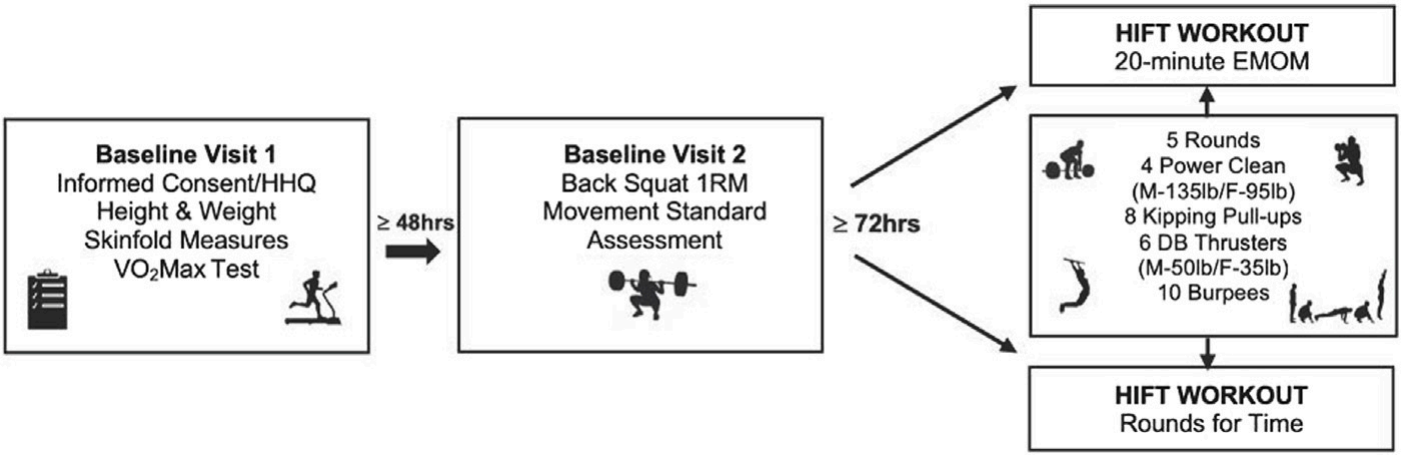
Summary of study design. Note: HHQ, health history questionnaire; HIFT, high-intensity functional training; M, male; F, female; EMOM, every minute on the minute; lb, pounds; DB, dumbbell; 1RM, one repetition maximum.

Baseline visit 1 was held at the University of New Mexico’s (UNM) Exercise Physiology Laboratory. Baseline visit 2 was followed by two randomized, balanced crossover HIFT trials (visits three and five), separated by at least 1 week, and were held at Big Barn CrossFit^®^ affiliate supervised by a CrossFit Level 2 (CF-L2) Trainer who is also an NSCA Certified Strength & Conditioning Specialist (CSCS). The order in which participants completed the HIFT trials was determined by a random number generator. Baseline visit one included anthropometrics and cardiorespiratory fitness (VO_2max_ measurement). Baseline visit two included a one-repetition maximum (1RM) lower body strength test (e.g., back squat) and an assessment of the movements of the prescribed exercises in the HIFT trials. For the HIFT trials, subjects performed two volume-matched HIFT workouts: (1) every minute on the minute (EMOM) and (2) “rounds for time” (RFT). Participants had pre- and post-exercise blood samples taken for measurement of blood lactate and creatine kinase (CK), a marker of muscle damage. Gas exchange was measured pre- and post-exercise to calculate excess post-exercise oxygen consumption (EPOC). HR, VO_2_, and RPE were measured during exercise (Figure 1).

### Participants

Participants were men (n = 6) and women (n = 6), 18–40 years of age, who had performed HIFT training for the last 12 months, with a minimum weekly frequency of 3 days per week. All participants completed a health history questionnaire, and individuals with any musculoskeletal injuries or limitations were excluded from the study. In the baseline visits, participants demonstrated their ability to meet the movement standards of the HIFT exercises and to perform the exercise protocols using the prescribed absolute loads. Participants were instructed to refrain from vigorous exercise for 24 h and caffeine and food for 4 h before baseline visits 1 and 2. In addition, participants were advised to abstain from caffeine and food for 4 h, alcohol for 24 h, and exercise for 48 h before the HIFT trials, and to maintain their regular daily diet during the study. The *a priori* sample size was calculated using G-power software (version 3.1.9.4) with an alpha level of 0.05 and power (1 − beta) of 0.80, and the number of participants required to make a valid analysis was N = 12. The variable with the lowest effect size (average heart rate) from a study using a similar methodology was used to guide the power analysis (Fernández-Fernández et al., 2015). Independent sample t-tests showed significant differences between women and men for body mass and height, but not for age, VO_2max_, HR_max_, relative back squat (BS) 1RM, and %BF (Table 1).

**TABLE 1:** Individual data between CrossFit^®^ trained men (n = 6) and women (n = 6).

Variable	Men (mean ± SD)	Women (mean ± SD)	*p*-value
Age (yr)	34 ± 4	29 ± 5	0.12
Height (cm)	180.6 ± 3.7	171.5 ± 6.4	0.01*
Body weight (kg)	88.4 ± 9.4	68.8 ± 10.4	0.01*
Body fat (%)	18.1 ± 5.8	17.4 ± 3.5	0.80
VO_2max_ (mL/kg/min)	48.3 ± 6.4	47.3 ± 5.7	0.77
HR_max_ (bpm)	184 ± 7	189 ± 12	0.46
Back squat 1RM (kg)	148.6 ± 32.3	99.9 ± 14.8	0.01*
Relative back squat 1RM (kg)	1.5 ± 0.1	1.7 ± 0.3	0.12

*Significant difference between men and women (*p* < 0.05); yr, year; cm, centimeters; kg, kilograms; VO_2max_, maximal oxygen consumption; mL, milliliters; min, minutes; HR, heart rate; bpm, beats per minute; RM, repetition maximum.

### Baseline visit 1

Upon arrival at UNM’s Exercise Physiology Laboratory, participants completed an informed consent and health history questionnaire. Then, participants’ heights (cm) were measured using a stadiometer (Holtain Limited, Crymych, Dyfed, Great Britain), and body weight (kg) was recorded using a digital weight scale (MedWeight MS-3900, Itin Scale Company, Brooklyn, NY, United States). Next, body composition was assessed via 3-site skinfold (SKF) measurements using a Lange skinfold caliper (Cambridge Scientific Industries, INC., Cambridge, Maryland). Two measurements within 1–2 mm were averaged. Three sites were measured: triceps, suprailiac, and thigh, and three sites were measured for men: chest, abdomen, and thigh (Jackson et al., 1980; Jackson and Pollock, 1978). Respective gender-specific equations were used to calculate body density (Jackson et al., 1980; Jackson and Pollock, 1978) and converted to a body fat percentage using the Siri equation (Siri, 1961). Finally, participants performed a maximal graded exercise test (GXT) on a treadmill (C966i, Precor Inc., Woodinville, WA, United States). In brief, treadmill speed was increased every minute by 0.5 mph, and once the top speed was reached, the percent grade was increased by 1% every minute until completion of the test. The initial speed was based on the participant’s self-selected top speed in the warm-up and a stage progression that allowed the participant to reach top speed at approximately 8 min. Gas analysis, measured breath-by-breath via a portable metabolic system (K5 wearable metabolic technology, COSMED, S.r.l., Italy), was used to determine VO_2max_. The criteria for establishing VO_2max_ were defined by reaching three of the four following parameters: reaching a plateau in VO_2_ of ≤150 mL O_2_/min, a maximal respiratory exchange ratio of >1.15, ±10 beats per minute (bpm) of the age-predicted maximum (220 − age), and RPE >17 (6–20 rating scale).

### Baseline visit 2

At the Big Barn CrossFit^®^ affiliate, maximal strength was tested via BS 1RM. The testing protocol included a warm-up set of 10 repetitions at 50% of BS predicted 1RM followed by 1 min rest; three repetitions with a 10%–20% increase in load followed by a 2-min rest; two repetitions with an additional 10%–20% increase followed by a 3-min rest; and a final 10%–20% increase for a one-repetition maximum attempt. Additional single repetitions, increasing in load, were performed with 3 min rest until the participant reached muscular failure (Haff and Triplett, 2016). The movement assessment included completing one round of the exercises prescribed in both HIFT protocols, including barbell power clean, kipping pull-up, dumbbell thruster, and burpee. During the performance of each exercise, the CrossFit Level 2 Coach (CF-L2)/Certified Strength & Conditioning Specialist (CSCS) professional visually inspected the participants’ ability to complete full range of motion (ROM) repetitions successfully with the prescribed absolute load. A description of the exercises is provided in the Supplemental Material.

### HIFT trial visits 3 and 5

The HIFT protocols were designed based on two different HIFT workouts: EMOM and RFT. Upon arrival at the Big Barn CrossFit^®^ affiliate, participants were seated in a chair for 10 min for baseline measurements. Prior to each workout, participants performed a 3-min general warm-up of low-intensity aerobic exercise (i.e., rowing ergometer), dynamic stretching, and a specific warm-up for the movements within the workout. In the EMOM trial, participants performed the following sequence of exercises: four barbell power cleans (minute 1), eight kipping pull-ups (minute 2), six dumbbell thrusters (minute 3), and ten burpees (minute 4). This sequence was repeated for a total of 20 min (five rounds). Once the repetitions were completed within the minute, participants rested for the remaining time of that minute. The prescribed exercises were based on common movements performed in HIFT that alternate between upper- and lower-body-dominant movements to balance fatigue and include major muscle group activation. The prescribed repetition scheme and load were selected to ensure participants would have approximately 30 s of rest after each exercise. The RFT trial included five rounds of the four exercises described above and was performed “all-out.” The subjects were encouraged to take minimal to no rest for the RFT. The velocity of the movements was not controlled; however, participants were asked to perform the movements as quickly as possible through the full range of motion. For both protocols, the prescribed absolute load for the barbell power clean and dumbbell thruster was 135 lb/95 lb and 50 lb/35 lb for males and females, respectively. A CF-L2/CSCS professional monitored both trials for all subjects to ensure each repetition was performed to the correct movement standards with a similar range of motion between the trials. Upon completion of the HIFT trials, participants returned to Big Barn CrossFit^®^ for visits 4 and 6 to complete their 24 h post-exercise blood draw.

### HIFT measurements

#### VO_2_, HR, and RPE

Oxygen consumption (VO_2_) was measured continuously throughout each protocol using a portable metabolic system (K5 wearable metabolic technology, COSMED, S.r.l., Italy). Participants wore a lightweight, slim-fitting vest that harnesses a sensor unit on their back to allow for proper performance of movements. A face mask, which includes a flow sensor, was attached to the sensor unit via a sampling line to collect expired gases. Participants were seated in a chair for 10 min prior to each HIFT trial for baseline VO_2_ as well as 20 min after exercise for recovery VO_2_. Baseline VO_2_ was determined using the average of the last 2 min of seated VO_2_ prior to the start of exercise (warm-up). The Polar RS800 heart rate chest strap was used to record the heart rate continuously for each trial. The VO_2_ and HR were averaged for each round and then averaged for each HIFT workout to determine the mean values. Average and maximum values of relative VO_2_ (mL/kg/min) were used to define participants’ VO_2peak_. EPOC was calculated from the VO_2_ data extracted from the metabolic analyzer using 10 s averaging to ensure the same time comparison between HIFT protocols. To determine the slow component of EPOC, the first 2 min of recovery VO_2_ data were excluded to remove data obtained during the transition from exercise to the seated position. The slow component EPOC was calculated by subtracting 18 min of the 20-min recovery VO_2_ from baseline VO_2_. For both HIFT trials, RPE was recorded at the end of each round using the modified category ratio 10 (CR10) RPE scale (Foster et al., 2001).

#### Blood sampling

Blood lactate (BLA) concentrations were determined from blood samples drawn from the earlobe and analyzed with a portable device (Lactate Plus Analyzer, Nova Biomedical, Waltham, MA) pre- and 3 min post-exercise. In addition, participants underwent venipuncture in a prominent forearm vein cleaned following standard sterile procedure, and blood serum samples were collected in BD Vacutainer™ Venous Blood Collection Tubes (Fisher Scientific, Carlsbad, CA) pre- and 24 h post-exercise. After coagulation, the samples were centrifuged at 2.900 RPM. The resultant serum was divided into several aliquots and frozen at −80°C until analysis. The pre- and 24 h post-exercise blood serum samples were sent to Quest Diagnostics for analysis of CK.

### Statistical analyses

All analyses were performed using GraphPad Prism Version 10.2.0 (335). The Shapiro–Wilk test was applied to verify a normal distribution of data, and Levene’s test was used to assess the homogeneity of variance. Dependent sample t-tests and effect size by Cohen’s *d* were used to compare time domains, mean HR, peak HR, mean HR percentage of HR_max_, peak HR percentage of HR_max_, mean VO_2_, peak VO_2_, mean VO_2_ percentage of VO_2max_, peak VO_2_ percentage of VO_2max_, mean RPE, peak RPE, and EPOC between the two workouts (EMOM and RFT). A two-way ANOVA with effect size by partial *eta* squared (ηp^2^) was applied to assess differences in and between resting and post-exercise BLA and CK. Lastly, two-way ANOVAs were performed to evaluate differences in VO_2_, HR, and RPE across the five rounds of exercise for both protocols. The level of statistical significance was set at *p* < 0.05.

## Results

Twelve participants completed this study, and their descriptive data are presented in Table 1. The prescribed HIFT exercises were well tolerated by all participants, and no injuries were reported.

The time to complete the RFT was significantly lower than the EMOM (t (11) = 9.39, *p* < 0.00, *d*_*z*_ = 2.69), as shown in Table 2. The results showed that individuals had an average of 49 s rest following the four power cleans, 45 s rest following the eight kipping pull-ups, 46 s rest following the six dumbbell thrusters, and 30 s rest following the burpees. The assessment procedures were well tolerated by all participants, and no injuries were reported.

**TABLE 2:** Comparison of variables between the two workouts (N = 12).

Variable	EMOM	RFT
BLA-Pre (mmol/L)	0.9 ± 0.2	0.9 ± 0.4
BLA-Post (mmol/L)	6.5 ± 2.7	11.2 ± 2.1**
VO_2avg_ (mL/kg/min) %VO_2max_	30.8 ± 3.0	38.1 ± 5.0**
	65 ± 7	80 ± 10**
VO_2peak_ (mL/kg/min) %VO_2max_	44.4 ± 5.2	48.3 ± 5.1*
	93 ± 8	102 ± 12*
HR_avg_ (bpm) %HR_max_	153 ± 19	171 ± 12*
	82 ± 7	91 ± 3*
HR_peak_ (bpm) %HR_max_	173 ± 13	182 ± 9*
	93 ± 4	98 ± 1*
Time domain (min:sec)	19:31 ± 0:09	11:48 ± 2:56**
RPE_avg_	4 ± 1	7 ± 1**
RPE_max_	5 ± 2	9 ± 1**

*Significant difference between workouts (*p* < 0.05), **(*p* < 0.001), WOD, workout of the day; EMOM, every minute on the minute; RFT, rounds for time; BLA, blood lactate; mmol, millimole; L, liter; VO_2avg_, average oxygen consumption; VO_2peak_, peak oxygen consumption; mL, milliliter; kg, kilogram; min, minute; HR_avg_, average heart rate; HR_peak_, peak heart rate; bpm, beats per minute; SmO_2_, muscle oxygen saturation; min, minutes; sec, seconds; RPE, rating of perceived exertion (modified category ratio 10 RPE scale).

### Metabolic, cardiorespiratory, and muscle damage responses

**FIGURE 2:**
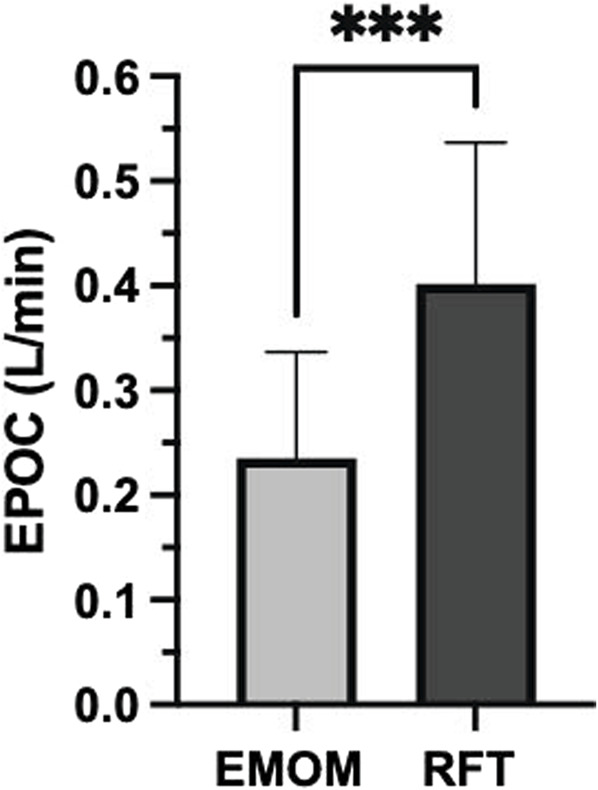
Excess post-exercise oxygen consumption (EPOC) calculated from 20 min of recovery for both conditions. L, liters; min, minute; EMOM, every minute on the minute; RFT, rounds for time. Significant differences between protocols (*p* < 0.05).

**FIGURE 3:**
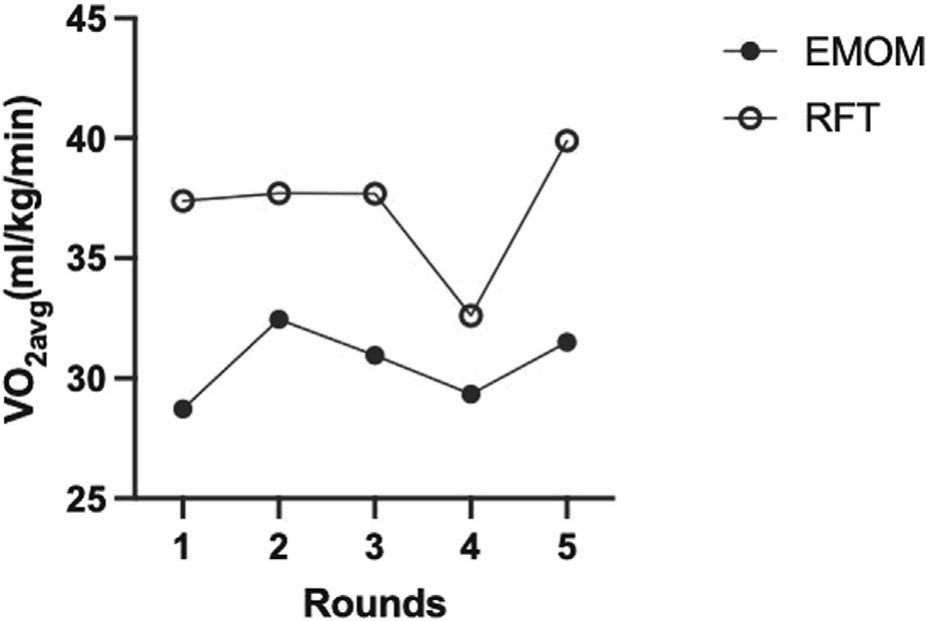
Average oxygen consumption (VO_2avg_) of all participants (n = 12) across all rounds of exercise for both protocols. EMOM, every minute on the minute; RFT, rounds for time.

**FIGURE 4:**
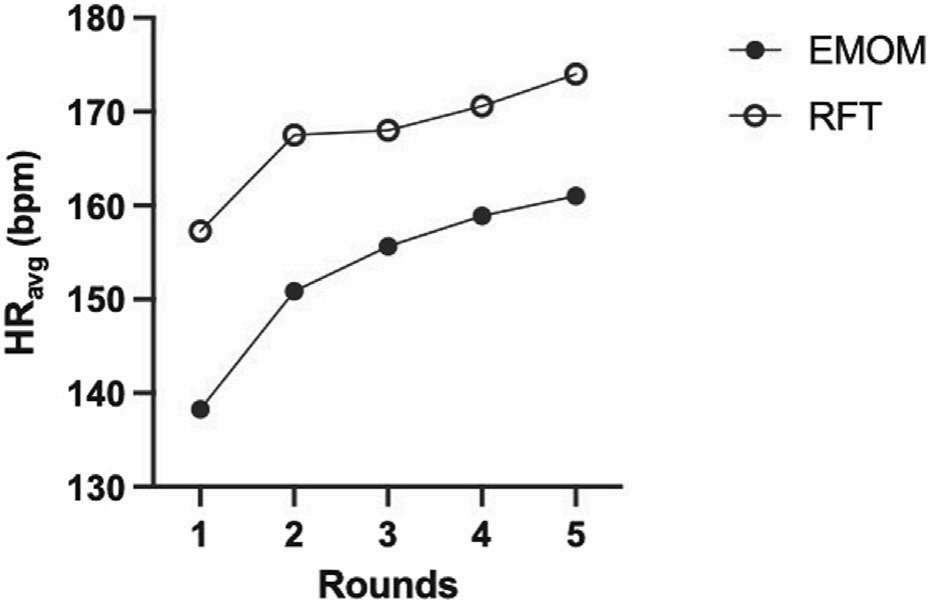
Average heart rate (HR_avg_) for the full sample (n = 12) across all rounds of exercise for both protocols. EMOM, every minute on the minute; RFT, rounds for time.

**FIGURE 5:**
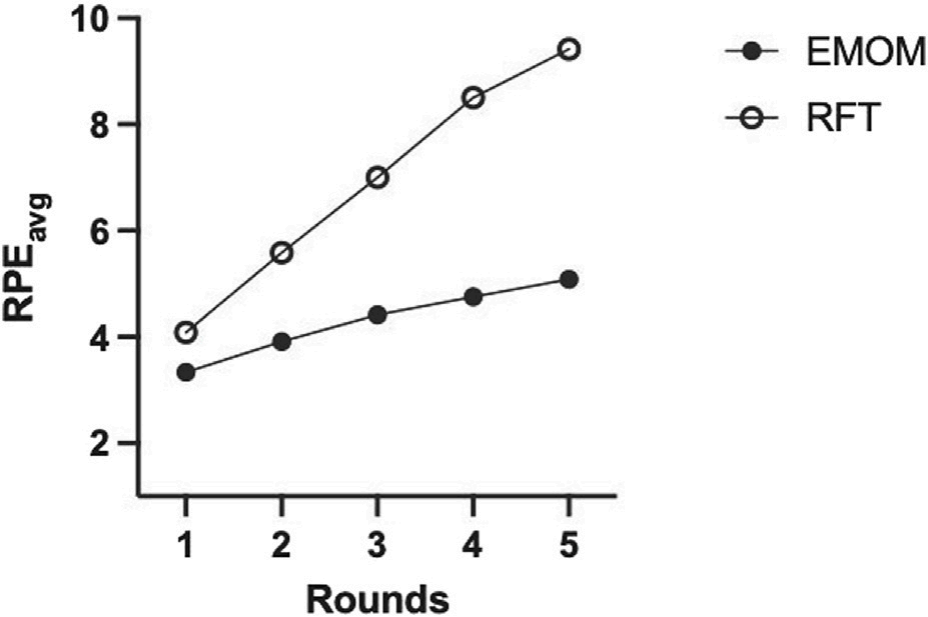
Average rating of perceived exertion (RPE_avg_) for the full sample (n = 12) based on a modified Borg scale of 1–10 across all rounds of exercise for both protocols. EMOM, every minute on the minute; RFT, rounds for time.

Descriptive statistics for both workouts are presented in Table 2. There were significant differences between the EMOM and RFT for time domains (t (11) = 9.39, *p* < 0.01, *d* = 2.78), mean HR (t (11) = 4.30, *p* < 0.01, *d* = 1.24), peak HR (t (11) = 4.41, *p* < 0.01, *d* = 1.27), mean VO_2_ (t (11) = 6.79, *p* < 0.01, *d* = 1.79), peak VO_2_ (t (11) = 3.15, *p* < 0.01, *d* = 0.76), mean VO_2_%, mean RPE (t (11) = 5.34, *p* < 0.01, *d* = 1.54), peak RPE (t (11) = 7.18, *p* < 0.01, *d* = 2.07), and EPOC (t (516) = 27.44, *p* < 0.01, *d* = 1.33) (Figure 2). The two-way ANOVA for VO_2_ and HR resulted in no interaction effect for time (rounds of exercise) and protocol (EMOM and RFT) (*p* = 0.16 and *p* = 0.37); however, there was a main effect for time (*p* = 0.00 and *p* < 0.00) and protocol (*p* = 0.00 and *p* = 0.05) (Figures 3, 4). Conversely, the two-way ANOVA for RPE showed an interaction (*p* < 0.00) and a main effect for time and protocol (*p* < 0.00) (Figure 5).

**FIGURE 6:**
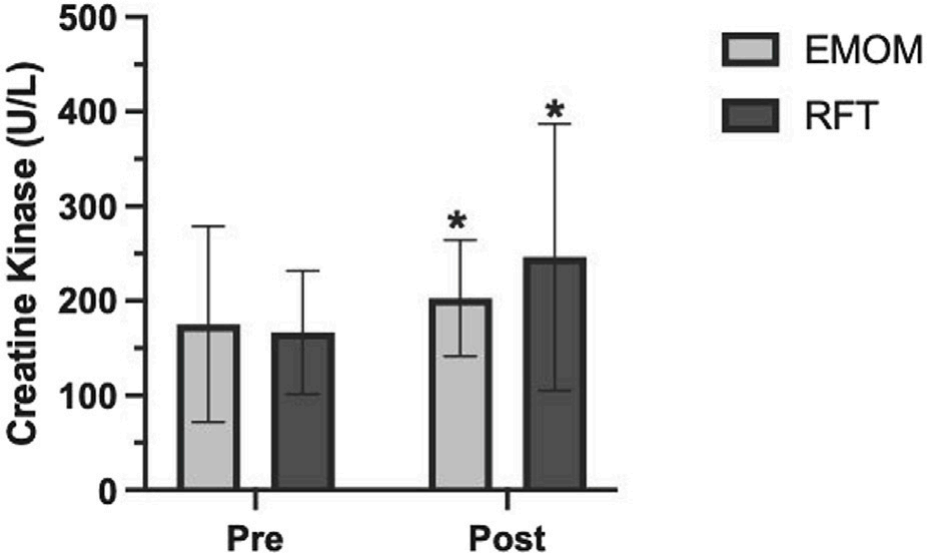
Creatine kinase pre-and 24 h post-exercise. U/L, units of enzyme activity per liter; EMOM, every minute on the minute; RFT, rounds for time. *Significantly different from baseline (*p* < 0.05).

Tukey’s multiple comparisons test was conducted to examine pairwise differences between EMOM and RFT across the five rounds of exercise. For the EMOM vs. RFT comparisons, significant differences were observed across all pairs (*p* < 0.05), with the largest difference in round 5 (mean difference = −4.333, 95% CI [-5.796, −2.871], *p* < 0.00). For within-EMOM comparisons, significant differences were observed between rounds 1 vs. 4, 1 vs. 5, 2 vs. 3, 2 vs. 4, and 2 vs. 5 (mean differences ranging from −0.583 to −1.750, *p* < 0.05). For within-RFT comparisons, significant differences were observed between all rounds, with the largest mean difference between round 1 and round 5 (−5.333, 95% CI [-6.162, −4.505], *p* < 0.00). Finally, the two-way ANOVA for BLA revealed a main effect for time (F (1, 11) = 191.6, *p* < 0.01, ηp^2^ = 0.95), indicating a significant increase from pre-to post-exercise across both workouts. Further, an interaction effect revealed BLA was lower for the EMOM than RFT post-exercise (F (1, 11) = 42.39, *p* < 0.00, ηp^2^ = 0.79). The two-way ANOVA mixed effects model for CK revealed a main effect for time (F (1, 11) = 5.14, *p* = 0.04, ηp^2^ = 0.32), indicating a significant increase from pre-to post-exercise across both workouts; however, there was no interaction effect (F (1, 9) = 3.86, *p* = 0.08, ηp^2^ = 0.30) (Figure 6).

## Discussion

In the present study, we compared the acute physiological responses of two different high-intensity functional workouts that were volume matched. One was an interval-based design with prescribed rest (EMOM), while the other was a continuous-based design performed “all-out” with self-selected rest (RFT). The main findings indicated that the RFT induced greater physiological stress, which is evident by higher values of VO_2,_ BLA, HR, EPOC, and RPE compared to the EMOM. Both workouts can be characterized as high-intensity exercise; however, when compared to the RFT, the EMOM may be considered more moderate intensity. Overall, both designs are comparable in intensity to other HIFT workouts reported in previous studies (Fernández-Fernández et al., 2015; Hernández-Lougedo et al., 2021; Maté-Muñoz et al., 2018; Toledo et al., 2021).

As previously mentioned, the workout was designed to ensure at least 30 s rest after each exercise in the EMOM, and the results showed that participants, on average, received at least 30 s of rest during the EMOM. The RFT took significantly less time (an average of 12 min) to complete the same amount of work than the 20-min EMOM. Although not statistically analyzed, it was observed that all participants were able to perform all repetitions for each movement consecutively in the EMOM, whereas some participants elected intra-set rest during the RFT. For example, the eight kipping pull-ups were performed in two sets of four repetitions with a brief rest period between. In addition, rather than performing four consecutive power cleans without releasing the bar, some participants opted to perform single repetitions. This can be attributed to the “all-out” nature of the RFT. Hernández-Lougedo et al., 2021 compared two protocols, both including a circuit of four rounds of exercises to be completed as quickly as possible (RFTstandard), but one of the protocols was adapted to include a 1-min rest interval after each round of exercise (RFTadapted). Unlike the present study, there was no significant difference in time to completion for the workouts, which may be attributed to more self-selected rest and reduced movement velocities in the RFT standard (Hernández-Lougedo et al., 2021). Relative work intensities may differ when including prescribed rest intervals for the same absolute training volume, including repetitions performed at a higher velocity, indicating a lower relative intensity for that absolute load and different ranges of effort and fatigue relevant to recovery of predominant energy systems (Hernández-Lougedo et al., 2021).

### VO_2_, HR, and RPE

Average VO_2_, percentage of VO_2max_, and EPOC were higher for the RFT than the EMOM protocol in the present study. Limited research has evaluated the metabolic demand of HIFT workouts via gas analyses, and no research, to our knowledge, has compared interval and continuous-based designs that are volume matched. Fernandez-Fernandez et al. (2015) characterized commonly prescribed HIFT workouts, including (1) 20-min AMRAP of five pull-ups, 10 push-ups, and 15 squats; (2) RFT of 21, 15, and 9 repetitions of barbell thrusters and pullups with an average completion time of 9 min. The 20-min AMRAP had a higher average VO_2_ and %VO_2max_ (34.4 ± 3.5 and 66.2 ± 4.8 mL/kg/min, respectively) than the RFT (29.1 ± 1.1 and 56.7 ± 6.2 mL/kg/min, respectively). Both AMRAP and RFT can be characterized as high-intensity workouts similar to the workouts in the present study. Regarding the effects of rest intervals on VO_2_, Haltom et al. (1999) compared two circuit weight training (CWT) protocols (matched for exercise selection and volume) that differed in rest intervals: one 20-s rest interval (20 RI) and one 60-s rest interval (60 RI). Their protocol included eight exercises: 1) leg press, 2) bench press, 3) leg extension, 4) lat-pull, 5) leg curl, 6) seated row, 7) triceps extension, and 8) biceps curl. They found that the 20 RI CWT protocol elicited a greater exercise VO_2_ and EPOC than the 60 RI CWT protocol. Although both protocols were interval-based and incorporated traditional resistance exercise rather than the functional exercise used in the present study, these results demonstrate that reduced rest periods induce greater metabolic stress.

With respect to heart rate responses, we found that %HR_max_ and average HR were significantly higher during the RFT than during the EMOM. However, a smaller difference occurred in the HR response (%HR_max_ ∼10% higher for RFT versus EMOM) than the VO_2_ response (%VO_2max_ ∼21% higher for RFT versus EMOM). The magnitude of the difference between the HR and VO_2_ responses may be due to the mechanical load and intramuscular pressure induced by skeletal muscle contraction during resistance-based exercise. This high intramuscular pressure generated during muscle contraction, especially in the multi-joint exercises used in the present study, temporarily occludes flow through the active muscles, increasing afterload and decreasing stroke volume. As a result, the heart must contract more to maintain cardiac output (Kounoupis et al., 2020).

Finally, the RPE (Borg CR10 scale) for the RFT is considered vigorous intensity, while the EMOM is moderate intensity (ACSM, 2022). The RFT RPE values are similar to other studies (Fernández-Fernández et al., 2015; Maté-Muñoz et al., 2018; R. Tibana et al., 2018; Toledo et al., 2021). Conversely, the EMOM resulted in lower RPE than the limited studies evaluating the effects of rest intervals that showed RPE values characterized as vigorous intensity (Hernández-Lougedo et al., 2021; Maté-Muñoz et al., 2018; R. Tibana et al., 2018). The EMOM included ≥30 s rest after each exercise, while previous studies prescribed less rest between exercises (e.g., 10 s) (Maté-Muñoz et al., 2018) or had prescribed rest after each round of a circuit of exercises (e.g., 1 min) (Hernández-Lougedo et al., 2021; Tibana et al., 2018).

### Blood lactate

Blood lactate significantly increased post-exercise for both protocols; however, the RFT elicited higher levels, suggesting a greater degree of effort, anaerobic contribution, and type II fiber recruitment during the RFT. Research has shown that continuous-based designs (RFT and AMRAP) elicit high BLA (>10 mmol/L) values in trained individuals, similar to the present study (Fernández-Fernández et al., 2015; Escobar et al., 2017; Maté-Muñoz et al., 2018; Tibana et al., 2018). Similarly, the limited studies that have evaluated interval-based protocols have shown BLA values of >10 mmol/L (Maté-Muñoz et al., 2018; Hernández-Lougedo et al., 2021); however, in the present study, BLA for the EMOM was <10 mmol/L, which may be attributed to the differences in workout design and rest interval prescription.

### Creatine kinase

The current study found an increase between CK levels pre- and 24 h post-exercise in both protocols, which is consistent with previous research (Tibana et al., 2019; Timón et al., 2019; Gomes et al., 2020). Both protocols involved a combination of upper and lower body movements performed as quickly as possible, which can significantly increase CK (Koch et al., 2014). There was no statistically significant difference in CK for the EMOM and RFT; however, CK showed an upward trend in the RFT, indicating that reduced rest periods during HIFT in trained individuals may have a greater effect on CK. While this is the first study to evaluate the effects of rest interval during HIFT on CK, research has shown CK levels are greater during resistance training performed with 1 min rest than 3 min rest between sets during traditional resistance training (Koch et al., 2014).

### Limitations

The present study has limitations that readers should consider while interpreting our results. Our sample size consisted of 12 participants (six males and six females), and we did not control for the timing of our female participants’ menstrual cycles. Although research suggests that exercise performance does not significantly change with menstrual cycle phases (McNulty et al., 2020), controlling for this variable would have reduced the potential impact of menstrual status on perceptual and physiologic responses. Also, it is important to acknowledge that the prescribed loads for the barbell power clean and dumbbell thrusters are common, absolute loads in HIFT and prescribed for practicality purposes rather than as a percentage of 1RM. Therefore, the prescribed weights used in the present study may have impacted individual relative effort.

## Conclusion

In conclusion, both the RFT and EMOM in the present study can be considered vigorous exercise (77–95% HR_max_ and 64–90% VO_2max_) according to ACSM’s estimated intensity for cardiorespiratory exercise (ACSM, 2022). When comparing the RFT and EMOM, the RFT elicited higher levels of metabolic stress indicative of potentially greater cardiorespiratory adaptations. In addition, the increases in blood lactate and creatine kinase from both protocols support a significant anaerobic contribution, suggesting a sufficient stimulus for muscular fitness adaptations (Kraemer and Ratamess, 2005; Wackerhage et al., 2019). The results of this study may provide insight into the proper application of an RFT and EMOM design within HIFT dependent on the desired training stimulus. Future studies on various HIFT workout designs with and without rest intervals to improve exercise prescription in trained individuals are highly warranted.

## Data Availability

The raw data supporting the conclusions of this article will be made available by the authors, without undue reservation.
