# Supplementary material for: The physiological responses to volume-matched high-intensity functional training protocols with varied time domains
Source: Front Physiol. 2025 Feb 11;15:1511961. doi: 10.3389/fphys.2024.1511961 (PMC11850382; doi:10.3389/fphys.2024.1511961)

**APPENDIX**

**MOVEMENT STANDARDS**

**1. Barbell Power Clean**

*Starting position:* Barbell begins on the ground in a deadlift position. Hips are above the knee but below the shoulders with neutral spine.


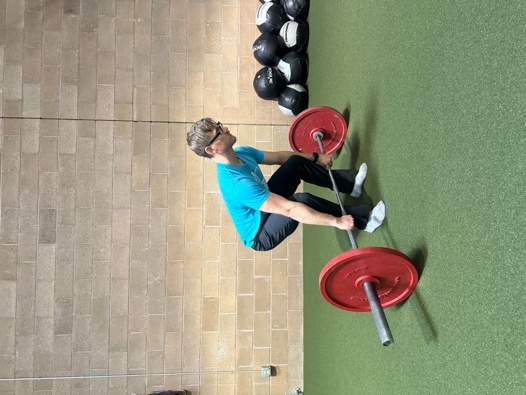


*Execution:* Knees and hips fully extend followed by elbow flexion and shoulder external rotation. Barbell comes up to the shoulders, received in a partial front squat finishing the lift with the hips and knees fully extended and the elbows in front of the bar.


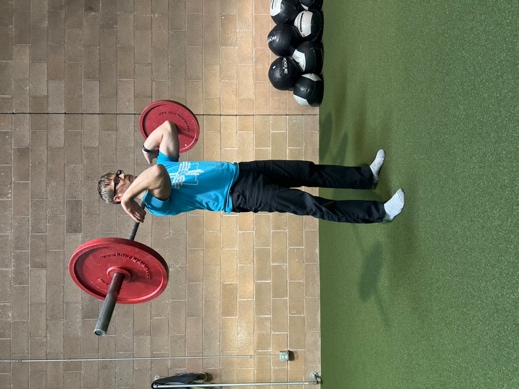

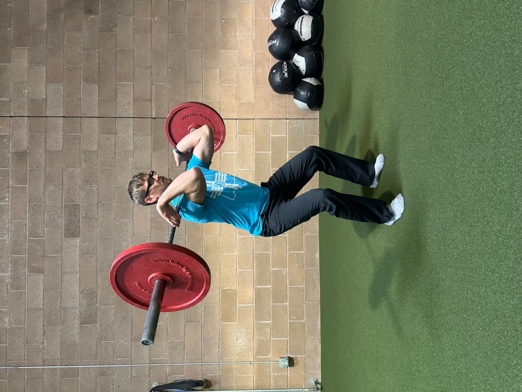


**2. Kipping Pull-up**

*Starting position:* Arms fully extended and feet off the ground.


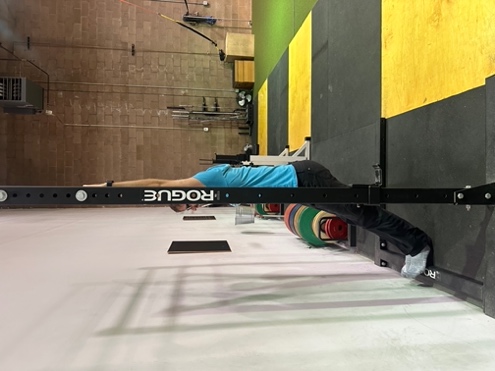


*Execution:* Swing the body forward into a “superman” position, push the body away from the pull-up bar into a “hollow body” position, then pull the body up to where the chin is over the bar.


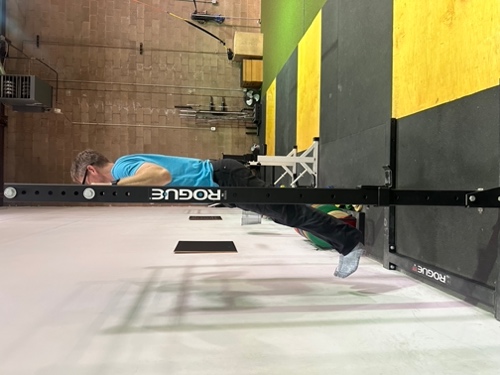


**3. DB Thruster**

*Starting position:* Fit are positioned between hip and shoulder width a part. Dumbbells positioned at anterior deltoids.

*Execution:* While maintaining a neutral spine, perform squat by hinging at the hips and flexing at the knees lowering to the point at which the crease of the hip is below the top of the knee. Fully extend the legs, hips, and arms pushing the dumbbells overhead.


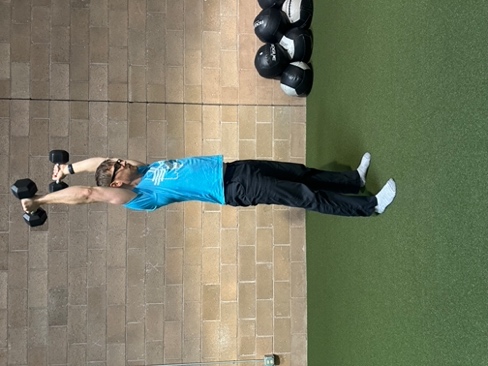

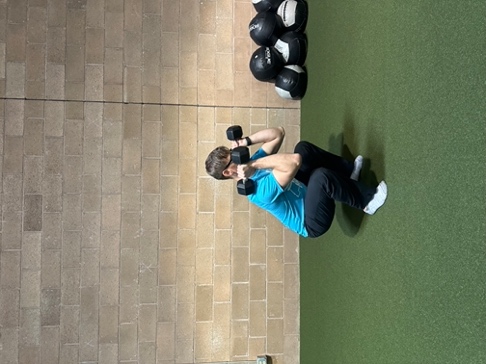


**4. Burpee**

*Starting position:* Stand fully extended.

*Execution:* Hinge at the hips and flex the knees to plant the palms on the floor and jump back lowering the body to the floor where the chest and thighs. Push body off the floor and thrust or jump forward landing in a squat position with feet flat on the floor. Jump to a fully extended position reaching hands overhead.


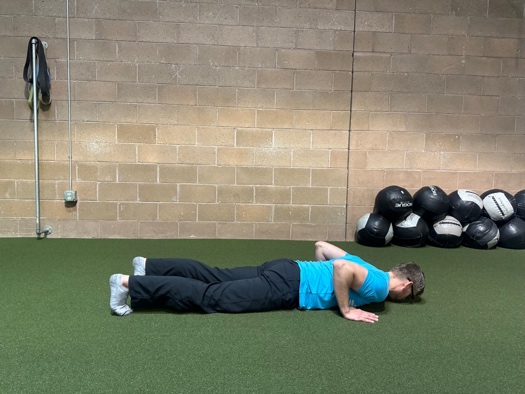


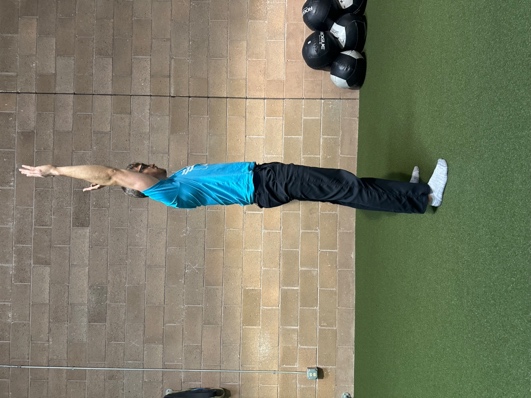

Supplement: Supplementary file 1 [file Table1.docx]
